# Notes and News

**Published:** 1902-12-27

**Authors:** 


### NOTES AND NEWS.

A consumption sanatorium has been opened at Baguley, Cheshire, by the Withington District Council, at a cost of £65,453.

A new wing has been opened at the Lynn Workhouse Infirmary. Two wards on the ground and first floors contain 37 beds each.

An isolation ward has been added to the Bradford Children's Hospital. It takes the form of a temporary pavilion, and has cost about £600.

The British Red Cross Society rendered splendid assistance during the war. They collected £180,213 in funds, and supplied an enormous amount of comforts to the sick.

The ward at the Cardiff Infirmary, provided by Mr. Thomas Andrews, has been opened. Funds for its maintenance, however, are not yet forthcoming for more than half its capacity.

An unfortunate result of utilising the Roentgen rays to remove superfluous hairs from the face is reported from Berlin, where a doctor has been fined £15, the patient's face having become red, and her lips swollen.

The new wing of the Kettering Hospital was opened by the Earl of Dalkeith on Saturday. Provision is made in it for a special ward for children and a new operating theatre. The Duke of Buccleuch gave the site, and the cost of the building was £8,000.

The Dudley Guest Hospital has received a benefaction, in a novel form, from Mr. H. Lewis, residing at Sutton Coldfield. An appeal was made for the hospital, and some time afterwards a registered envelope, containing £500, was received, followed by the arrival of seven van-loads of expensive furniture. The donor would not permit of the sale of the furniture, and stipulated in what direction the cash should be applied.

Dr. Garnault's report on his experiments to prove the fallacy of Professor Koch's contention that bovine tuberculosis is not communicable to human beings has now been completed. The experiments lasted from July to November, and all that Dr. Garnault appears to have established is that the infection did not in his case extend widely.

The Delamere Forest Sanatorium for Liverpool patients, the plans of which we published in a recent issue, has met with a generous friend in Mr. W. P. Hartley, the chairman of the institution. The need for the sanatorium and for still further accommodation in it is so patent that he has offered to provide 20 more beds in a wing which will cost probably £10,000 when complete. Mr. Hartley contributed about £7,500 towards the original building.

The Countess of Ilchester opened last week the Princess Christian Hospital and Sanatorium at Weymouth. The new institution succeeds the small building which was founded in 1848, and which was increased in size on several subsequent occasions. The hospital is arranged in one block. There are four large wards, for 12 beds in each, and small single wards are provided on first and second floors. There are day and dining rooms for convalescents. Operating theatre and suitable arrangements for out-patients are included.

A wing has been added to the Retford Hospital, containing a ward constructed as a memorial to the late Miss Gylby, the cost being defrayed by her neices Mrs. H. Maxfield and Mrs. Annie Hutchinson, and another ward for children given by Mrs. S. Smith as a memorial to her sister, Miss Mee, of Hayton. The amount expended on the wing amounts to about £800, but there is still a good deal to be done for which money is required. Sir F. Milner has given £100 to endow a cot in the children's ward in memory of his late wife.

The Police Seaside Home, Hove, Brighton, provides for all policemen needing a rest and change of air, with good food and home comforts after sickness or injury. The present home was opened in 1893, the foundation stone having been laid by H.R.H. Princess Christian. Since its establishment upwards of 7,000 men have been received from the Metropolitan and other police forces, most of whom have returned well and strong to their duty. The home is at present in great need of support, over £400 being urgently needed to meet liabilities.

The Worcestershire Sanatorium for Consumptives, situated on the Ankerdine Hills has been opened. It consists of a house, practically provided rent free by Mr. J. Dangerfield, to which open-air annexes have been added. It provides 16 beds 10 of which are free. An assured annual income of £800 only has been secured. Annual subscribers are entitled to the use of a free bed for patients sent by them, and several firms and public bodies have taken advantage of the offer, which will certainly afford them remarkably economical means of benefiting their employés.

